# Effect of an EMG–FES Interface on Ankle Joint Training Combined with Real-Time Feedback on Balance and Gait in Patients with Stroke Hemiparesis

**DOI:** 10.3390/healthcare8030292

**Published:** 2020-08-24

**Authors:** Subeen Bae, Jin Lee, Byoung-Hee Lee

**Affiliations:** 1 Graduate School of Physical Therapy, Sahmyook University, Seoul 01795, Korea; baesubeen@naver.com (S.B.); leejin87@hanmail.net (J.L.); 2 Department of Physical Therapy, Sahmyook University, Seoul 01795, Korea

**Keywords:** stroke, action observation, real-time feedback, balance, gait

## Abstract

This study evaluated the effects of an electromyography–functional electrical stimulation interface (EMG–FES interface) combined with real-time balance and gait feedback on ankle joint training in patients with stroke hemiplegia. Twenty-six stroke patients participated in this study. All subjects were randomly assigned to either the EMG–FES interface combined with real-time feedback on ankle joint training (RFEF) group (*n* = 13) or the EMG–FES interface on ankle joint training (EF) group (*n* = 13). Subjects in both groups were trained for 20 min a day, 5 times a week, for 4 weeks. Similarly, all participants underwent a standard rehabilitation physical therapy for 60 min a day, 5 times a week, for 4 weeks. The RFEF group showed significant increases in weight-bearing lunge test (WBLT), Tardieu Scale (TS), Timed Up and Go Test (TUG), Berg Balance Scale (BBS), velocity, cadence, step length, stride length, stance per, and swing per (*p <* 0.05). Likewise, the EF group showed significant increases in WBLT, TUG, BBS, velocity, and cadence (*p* < 0.05). Moreover, the RFEF group showed significantly greater improvements than the EF group in terms of WBLT, Tardieu Scale, TUG, BBS, velocity, step length, stride length, stance per, and swing per (*p* < 0.05). Ankle joint training using an EMG–FES interface combined with real-time feedback improved ankle range of motion (ROM), muscle tone, balance, and gait in stroke patients. These results suggest that an EMG–FES interface combined with real-time feedback is feasible and suitable for ankle joint training in individuals with stroke.

## 1. Introduction

Stroke can damage physical or mental abilities, depending on the location and size of the impaired area. It also often results in motion disorders. Approximately 20% of stroke survivors show foot drop. This affects walking and other daily activities [1].

Foot drop is caused by weakness or lack of spontaneous control in the ankle and toe dorsiflexors [2]. It can reduce the speed during walking and cause shifting or confusion while embracing one’s body weight. Furthermore, foot drop may result in inefficiency and instability during walking [3]. Foot drop limits the appropriate toe clearance during the swing phase. It also decreases the affected lower limb’s weight support down to 43%, increasing the risk of falling due to loss of balance [4]. Arbitration methods for improving the balance and walking abilities of stroke patients include motion observation [5], virtual reality [6], feedback [7], functional electrical stimulation [8], and task-oriented [9] training sessions. Functional electrical stimulation (FES) training improves the performance of daily activities [10] by increasing muscle strength and preventing muscle atrophy and rigidity [11] by electrically stimulating weak and paralyzed muscles. However, FES is an open-chain controlled system that contracts muscles with passive stimulation. In contrast, an electromyography–functional electrical stimulation interface (EMG–FES interface) training requires active patient intervention. Active FES training is more effective than passive in restoring the function of stroke patients [12].

The rehabilitation of stroke patients does not entail a new functional movement. Rather, it involves a process of rediscovering learned movements and relies heavily on motion-imaging techniques and motion observation training [13]. Motion observation training is a method that activates the motor cortex and mobilizes mirror neurons when performing actions and observing images, the purpose of which is to promote functional reconstruction in the brain of stroke patients [14]. Exercise learning through motor observation leads to skilled movements and permanent changes associated with exercises and experiences. The mirror neurons are more likely to be activated when a movement is observed more closely, such as in observing target-oriented related movements rather than a simple movement [15]. Real-time feedback enables the recognition and correction of motion errors during body movements to perform more effective functions. It also enables the learning of new and better ankle joint flexion in stroke patients [16].

Previous studies conducted physical training only after a certain period of action observation [5,14]. A study combining action observation with real-time feedback is scarce [15]. In addition, stroke patients may have cognitive problems that decrease their memory, concentration, and thinking abilities [17]. Thus, a study determining the effectiveness of ankle training combined with muscle conductive functional electrical stimulation is needed because this approach simultaneously provides the stroke patient with both exercise learning by intensive movement observation of the lower body and live feedback.

## 2. Materials and Methods

### 2.1. Participants

This study included 26 hemiplegic stroke patients at M rehabilitation hospital in Seoul. All participants in this experiment agreed after hearing the explanation of the progress, purpose, and possible side effects of the experiment. Before recruiting participants for this study, we performed a power analysis using G*Power version 3.1.9.7 (Heinrich-Heine-Universität, Düsseldorf, Germany). The overall effect size index for all the outcome measures and power of the study were 0.58 and a probability of 0.05 in order to minimize type II error (power of 80%). Because the estimated target sample size was 26, we recruited 30 participants for this experiment. The inclusion criteria for subject selection were hemiplegic stroke patients with a mini-mental state examination-Korean (MMSE-K) score higher than 24, no visual loss or impairments, ability to walk more than 10 m with or without ancillary tools, ankle plantar flexor muscle tone more than the fair grade, and dorsiflexion ankle muscle tone less than the Modified Ashworth Scale (MAS) grade 2. The exclusion criteria were inability to understand the oral instructions because of visual defects or severe visual impairment, cognitive impairment, serious cardiovascular disorders due to a severely damaged coronary artery, or serious limitation of passive joint movement due to orthopedic damage.

The present study was approved by the Myung-Ji Choon-Hey Hospital Institutional Review Board (MJCHIRB-2016-02) in Republic of Korea. The objective and the procedures to be performed in the study were fully understood by the subjects, and all subjects provided informed consent for inclusion in the study. Therefore, this study was based on the ethical principles of the Declaration of Helsinki.

### 2.2. Experimental Procedures

**Figure 1:**
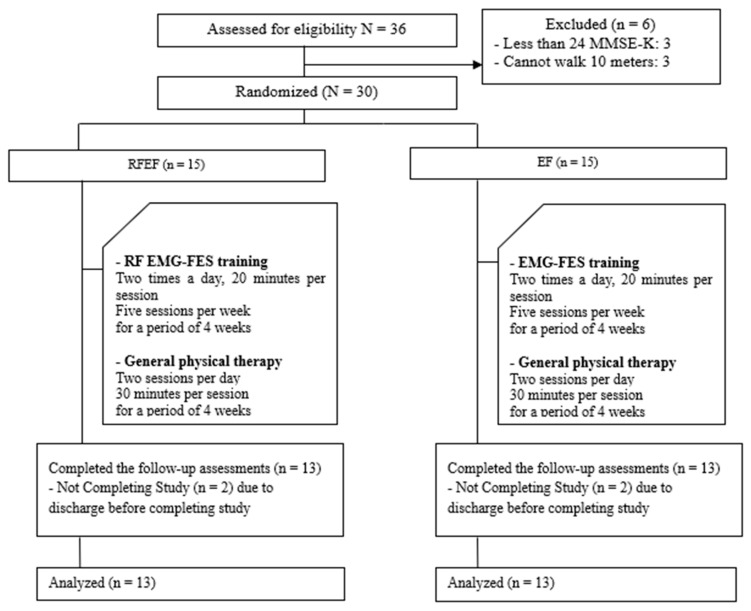
Schematic of the study design. Real-time feedback (RF) electromyography–functional electrical stimulation interface (EMG–FES interface) = EMG–FES interface combined with real-time feedback on ankle joint training.

The participants were randomly divided into two groups one hour before the start of training to minimize errors caused by group selection. All subjects picked up a black or white stone from a box containing 30 pieces of stone. The participants were divided into the EMG–FES interface combined with real-time feedback on ankle joint training (RFEF) group (*n* = 13) and EMG–FES interface on ankle joint training (EF) group (*n* = 13). Pre- and post-evaluation assessments were performed one week before and after training, while the test itself was conducted over a four-week period. The RFEF group had training sessions twice a day at 20 min duration each. Combined real-time feedback and EMG induction functional electrostimulation therapies were performed 5 times a week over a span of 4 weeks. The EF group, however, performed only EMG induction functional electrostimulation therapy under the same conditions. Both RFEF and EF groups performed general physical therapy twice a day at 30 min duration each, 5 times a week, for 4 weeks (Figure 1).

### 2.3. Intervention

#### 2.3.1. Real-Time Feedback

Real-time feedback allowed participants to sit on chairs with armrests and watch 27-inch TV screens installed 2 m in front of them in a comfortable state. A motion observation screen and a real-time feedback screen were displayed on one monitor at the same time and provided at the participants’ eye level. The motion observation screen showed a model with no problem with the ankle dorsiflexion and the motion observation video was played to match the electrical duration of the subject’s EMG–FES interface through a TV screen connected to the laptop. The real-time feedback was set up to film the participants’ ankle from the side with a web cam and show them on the screen in real-time. Before performing combined real-time feedback and EMG functional electrical stimulation for ankle joint training on all subjects, directive verbal cues were given, such as “Lift your ankle according to the movement observation screen” and “Follow the instructions as you modify the way you raise your ankle”.

#### 2.3.2. EMG–FES Interface Training

EMG–FES interface is a biofeedback electrotherapy device that combines the patient’s own power with the function of functional electrostimulation therapy, designed to stimulate electricity and contract muscles when the patient themselves applies a force above a certain threshold. To reduce the electrical skin resistance of subjects, the skin of the tibialis anterior muscle was wiped with an alcohol swab, and an active electrode was attached to the proximal part of the tibialis anterior muscle, a reference electrode was attached to the distal part, and an EMG electrode was attached to the center between the active and reference poles. The active electrode is attached to the area that responds most strongly to the electrical stimulation. Before beginning the experiment, the investigator passively modulated the stimulated current intensity frequency 35 Hz and pulsed with 250 μs from 1 mA to 50 mA according to the response from the subject’s ankle joint. The threshold was set according to the voluntary muscle contraction for each patient. When the set threshold is exceeded, the electrical stimulation is automatically initiated so that the electricity is stimulated by a 0.1 s rise, a 5 s on, and a 2 s decay. Before performing EMG functional electrical stimulation for ankle joint training on all subjects, directive verbal cues were given, such as “Lift your ankle according to the movement observation screen” and “Follow the instructions as you modify the way you raise your ankle”.

#### 2.3.3. General Physical Therapy

General physical therapy was performed by a physical therapist once or twice for 30 min. The Proprioceptive Neuromuscular Facilitation (PNF) technique, neurodevelopmental treatment, and Bobath therapy were carried out twice a day for 30 min each. The same therapy was performed on all subjects by the same physical therapist for 5 sessions weekly over a duration of 4 weeks, and for 4 weeks on all subjects by the same physical therapist. Each method was used for the subjects of this study.

### 2.4. Outcome Measurement

The weight-bearing lunge test (WBLT) was used to measure the ankle’s range of motion (ROM). The Tardieu Scale (TS) was used to assess muscle tone, while the Timed Up and Go Test (TUG) and the Berg Balance Scale (BBS) were used to measure balance. Additionally, GAITRite™ (CIR systems, Inc., Franklin, NJ, USA, 2008) was used to measure the time–space gait variables.

The WBLT is conducted with both feet perpendicular to the wall and lunge motions directed toward the wall. The maximum range of ankle plantar flexion was the range at which the heel came in contact with the floor. At this point, the ankle joint range measurement was recorded, along with the distance from the foot to the wall. The WBLT has a high confidence (*r* = 0.97–0.98) based on the measurer of the assessment and the confidence between the measurers (*r* = 0.97–0.99) [18].

The Tardieu Scale was used to measure muscle tone. To measure the relative strength of the gastrocnemius muscle (GCM), the subject’s knees were bent by more than 30° while in the supine position. The ROM of the subject’s ankles was measured at the lowest possible rate at which the muscular strength was measured [19]. The high confidence in the measurers (*r* = 0.87) and between measurements (*r* = 0.91) of the Tardieu Scale makes it suitable for the assessment of muscle strain [20].

The TUG was used to evaluate balance. [21] It was performed by measuring the time from being seated on a chair with armrests, standing at the command of “Start”, walking to the front three meters away, making a turn to come back, and sitting on the same chair. The TUG is a reliable tool with a high level of confidence in the assessment of the measurers (*r* = 0.99) and between measurements (*r* = 0.98) [22].

The BBS was used to measure static and dynamic balancing capabilities. The test required performing each item on a 14-part scale (minimum, 0; maximum, 4; total, 56). For the BBS, a higher score corresponded to better balance with a high level of confidence in the measurers (*r* = 0.99) and between measurements (*r* = 0.98) [23].

The GAITRite device was used to measure the time and space walking variables of stroke patients who were made to traverse a walkway 61 cm wide and 366 cm long. The special sensors on the walkway guaranteed measurement of the subject’s walking velocity and cadence as well as space-related characteristics such as step and stride lengths. GAITRite is a reliable tool with a high level of confidence in the measurers (*r* = 0.72–0.94) and between measurements (*r* = 0.81–0.99) [24].

To minimize the measurement error of dependent variables in this study, one examiner and one assistant performed the inspection before and after the training. Average values were obtained by measurements taken thrice after one practice. A break period of 1 min between measurements was given to minimize fatigue.

### 2.5. Data Analysis

All statistical analyses were performed using SPSS version 22.0 (IBM Co., Armonk, NY, USA). Measurements were expressed as mean and standard deviation. All of the participants were tested for normality through the Shapiro–Wilk test. As a result, the data of the subjects had a normal distribution. Descriptive statistics were used to assess the participants’ general characteristics. The independent *t*-test was used to investigate the difference between groups. A paired *t*-test compared the before–after results between the RFEF and EF groups. The statistical significance level for all the data gathered was set at 0.05.

## 3. Results

A total of 26 subjects participated in this study. The demographic characteristics of all subjects are shown in Table 1. All the general characteristics of the subjects showed normality and homogeneity.

The RFEF group had 13 subjects, 5 (38.5%) of whom were male and 8 (61.5%) were female. The subjects had an average age of 57.69 ± 9.49 years, average height of 163.69 ± 10.26 cm, average weight of 60.23 ± 11.27 kg, and average body mass index (BMI) of 22.41 ± 3.28 kg/m^2^. The subjects of the EF group comprised 5 males (38.5%) and 8 females (61.5%). They had an average age of 49.15 ± 12.80 years, average height of 162.46 ± 11.98 cm, average weight of 60.85 ± 9.88 kg, and average BMI of 23.03 ± 2.44 kg/m^2^. There was no significant difference between the groups.

### 3.1. Lower-Limb Function

As a result of evaluating ankle joint ROM, the RFEF group increased significantly from 3.30 cm to 5.23 cm before training (*p* = 0.000), and the EF group increased significantly from 3.65 cm to 4.38 cm before training (*p* = 0.000). Comparing differences between groups resulted in statistically significant differences (*p* = 0.015). In muscle tone, the RFEF group significantly decreased from 1.35 points to 0.79 points before training (*p* = 0.003), and the EF group decreased from 1.17 points to 1.06 points before training, but there was no significant difference. Comparing differences between groups resulted in statistically significant differences (*p* = 0.025) (Table 2).

### 3.2. Balance

As a result of evaluating the TUG, the RFEF group significantly decreased from 36.54 s to 28.73 s before training (*p* = 0.004), and the EF group significantly decreased from 20.84 s to 18.49 s before training (*p* = 0.033). Comparing differences between groups resulted in statistically significant differences (*p* = 0.035). The BBS assessment significantly increased for the RFEF group from 37.38 to 44.53 points before training (*p* = 0.000), and its value for the EF group increased significantly from 40.69 to 44.69 points before training (*p* = 0.001). Comparing differences between groups resulted in statistically significant differences (*p* = 0.032) (Table 3).

### 3.3. Gait Ability

As a result of evaluating the gait speed in walking ability, the RFEF group increased significantly from 38.85 cm/sec to 49.73 cm/sec before training (*p* = 0.001), and the EF group increased significantly from 52.47 cm/sec to 56.81 cm/sec before training (*p* = 0.021). Comparing differences between groups resulted in statistically significant differences (*p* = 0.044). In the cadence, the RFEF group increased significantly from 69.62 steps/min to 76.60 steps/min before training (*p* = 0.031), and the control group increased significantly from 75.62 steps/min to 78.68 steps/min before training (*p* = 0.038). There were no statistically significant differences in the comparison of differences between groups (Table 4).

As a result of evaluating the step length of the paretic leg in gait ability, the RFEF group increased significantly from 31.83 cm to 37.69 cm before training (*p* = 0.000), and the EF group increased significantly from 38.81 cm to 40.49 cm before training, but there was no significant difference. Comparing differences between groups resulted in statistically significant differences (*p* = 0.016). In the stride length of the paretic leg, the RFEF group increased significantly from 60.01 cm to 70.35 cm before training (*p* = 0.000), and the EF group increased significantly from 78.67 cm to 82.17 cm before training, but there was no significant difference. Comparing differences between groups resulted in statistically significant differences (*p* = 0.020). At the stance phase rate of the paretic leg, the RFEF group significantly decreased from 74.70% to 70.30% before training (*p* = 0.010), and the EF group decreased from 72.12% to 71.06% before training, but there was no significant difference. Comparing differences between groups resulted in statistically significant differences (*p* = 0.050). As a result of evaluating the swing phase rate of the paretic leg, the RFEF group increased significantly from 25.28% to 29.70% (*p* = 0.010) before training, and the EF group increased significantly from 27.88% to 28.94% before training, but there was no significant difference. Comparing differences between groups resulted in statistically significant differences (*p* = 0.049) (Table 4).

## 4. Discussion

Foot drop is one of the motor functions affected by deformed neural transmission and reduced active control of the feet during gait. Patients without ankle dorsiflexion tend to drag their feet while walking and show a gait abnormality similar to that in lifting their hips to prevent the toes from touching the floor. This foot drop causes gait instability and may lead to fractures from falls [25].

Using the WBLT, this study assessed the ankle joint ROM before and after training. Ankle joint training involving real-time feedback and EMG-triggered FES was performed 20 min a day, 5 times a week, for 4 weeks. As a result, the RFEF group had a significant increase of 1.92 cm, from 3.30 cm initially to 5.23 cm. The EF group also had a significant increase of 0.73 cm, from 3.65 cm initially to 4.38 cm. The groups were significantly different (*p* = 0.015).

In the research conducted by Chung et al. (2015) [26], the brainwave-based brain–computer interface (BCI)-FES system was applied to ten stroke patients who had five sessions of training (1 h per session) for ankle dorsiflexion. As a result, the experimental group experienced significantly improved TUG, cadence, and step length on the affected side after training (*p* < 0.05). The ankle joint ROM helps in adaptation to various kinds of floors and functions in shock absorption to increase stability while standing and walking. Stroke patients have limited ankle joint ROM, which directly and negatively influences functional ability [27].

FES treatment has been known to enhance muscle strength, prevent muscle weakness, and improve ROM [8]. However, simple repeated FES without a stroke patient’s active intervention is less effective in motion relearning, which is important for the patient’s recovery. EMG-triggered FES is more effective than general FES [12]. In the passive motion made by simple repeated electrical stimulation, there is no concentration of cognitive function and mobilization of the efferent motor nerve. In EMG-triggered FES, an increase in continuous concentration to move the muscles causes the activation of motor function. In sensory integration theory, the intent to move target muscles, a somatic sense feedback by the motion of target muscles, and the transmission of electrical stimulation are integrated in the cerebrum simultaneously or sequentially [28]. According to Begg et al. (2014) [16], real-time feedback application helped stroke patients find and correct errors of ankle motion. This promoted passive re-education and consequently improved the gait ability. Thus, if multiple stimulations were used for motor function training, these would more significantly influence brain plasticity than simple stimulation [29]. In this study, the improvement of the ankle’s ROM was attributed to the muscle strength enhancement and muscle tension relaxation brought by the combined effects of FES and the advantages of EMG-triggered FES training for inducing active muscle activities. In addition, by allowing the patient to correct an ankle joint motion error on their own through the real-time feedback during training, it was possible to improve the ankle ROM.

Muscle tone pertains to muscle resistance in the manual stretch during resting and the continuously passive and partial contraction of muscles while maintaining posture [30]. Using the Tardieu Scale, this study assessed changes in muscle tension before and after training. Ankle joint training with real-time feedback and EMG-triggered FES was performed 20 min a day, 5 times a week, for 4 weeks. As a result, the experimental group had a significant decrease of 0.55 points, from 1.35 initially to 0.79. There was a significant difference between groups (*p* = 0.003).

In the research performed by Zhu et al. (2015) [31], a test was conducted among 61 stroke patients at 30-min durations for each session, with 6 sessions weekly for 8 weeks. The experimental group had motion observation training, daily rehabilitation, and nursing, while the control group had daily rehabilitation and nursing only. As a result, the Modified Ashworth Scale (MAS) score of the experimental group significantly decreased from 2.71 to 1.65 points, whereas that of the control group decreased from 2.53 to 2.07 points. In the research conducted by Yom et al. (2015) [32], a test was conducted on 20 stroke patients at 30-min durations for each session, with 5 sessions weekly for 6 weeks. The experimental group had a virtual reality-based ankle motion, whereas the control group watched a video. The MAS score of the experimental group significantly decreased by 0.90 points, from 1.67 to 0.75, whereas that of the control group decreased by 0.10 points, from 1.70 to 1.60. On the Tardieu Scale, the MAS score of the experimental group decreased by 1.10 points, from 2.20 to 1.10, whereas that of the control group decreased by 0.20 points, from 2.40 to 2.20. The groups had a significant difference (*p* < 0.05).

In terms of virtual reality’s advantages in improving muscle tension, the virtual reality-based ankle and lower extremity motion was effective at improving stroke patients’ gait speed, ankle movement, and soleus muscle strength [33]. Pathological muscle tension reduction, one of the positive symptoms of upper motor neuron syndrome, leads to stretch reflex reduction through ankle movement. This causes an increase in the threshold of the phasic stretch reflex and a decrease in the tonic stretch reflex. The reaction results in a reduced muscular contraction reaction and increased ankle movement [34]. Accordingly, the tibialis anterior contraction and the gastrocnemius stretch cause muscle tension. For these reasons, training with real-time feedback and EMG-triggered FES was effective in improving muscle tension reduction. This study, however, differs from previous studies in terms of the evaluation method.

Damage to balance after stroke reduces the stability of posture while standing and causes difficulty with gait and functional activities. Therefore, the ability to balance is based on independent motions and functional activities. A stroke patient’s gait reduction is deeply related to balance damage [35]. For this reason, improving balance is key to improving a stroke patient’s gait. Balance is an essential factor for the execution of various motions in the activities of daily living or optimal gait function [36]. Using the TUG and the BBS, this study assessed changes in balance before and after training. The TUG was used to evaluate and quantify the functional mobility and dynamic balance with a time lapse. A clinical change was determined, and the reliability and validity of the TUG were approved [37]. The TUG for ankle joint training was performed 20 min a day, 5 times a week, for 4 weeks. As a result, the RFEF group had a significant decrease of 7.81 s, from 36.54 to 28.73 s, whereas the control group had a significant decrease of 2.35 s, from 20.84 to 18.49 s. There was a significant difference between groups (*p* = 0.035).

Park et al. (2015) [38] conducted a test on 40 stroke patients. The experimental group had action observation-based gait training 5 times a week, for 8 weeks, whereas the control group had general gait training for the same period. In terms of the TUG, the experimental group had a significant decrease from 20.0 to 15.7 s, whereas the control group had a significant decrease from 20.5 to 17.7 s. Comparing the TUG pretest values of this study with those of other studies, the RFEF group of this study obtained 36.54 s, compared with the results of Osaka et al. (2017) (12 s), Park et al. (2015) (20 s), and Ng et al. (2005) (22.6 s), indicating that they are functionally more severe participants. Nevertheless, we believe that this is because the participants’ MMSE-K score was 27.54, which indicates mild cognition impairment, and the Tardieu Scale of 1.35 points means that muscle tone was not high.

On the BBS, as a result of the ankle joint training involving real-time feedback and EMG-triggered FES 20 min a day and 5 times a week, for 4 weeks, the experimental group had a significant increase of 7.15 points (from 37.38 to 44.53 points), whereas the control group had a significant increase of 4.00 points (from 40.69 to 44.69 points). The two groups had a significant difference (*p* = 0.032).

In the research performed by Robertson et al. (2010) [39], they conducted a test on 15 stroke patients. The experimental group with the FES attachment had balance and mobility training for 2 h weekly for 4 weeks, whereas the control group had balance and mobility training during the same period without the FES attachment. As a result, on the BBS, the experimental group increased to 47.9 points, whereas the control group increased to 46.7 points.

Stroke patients have an increased risk of falls due to difficulties in balancing and abnormal muscle tension. The latter influences asymmetric postures such that it reduces a stroke patient’s sensory input and impedes proper body support upon assuming a standing posture. Applying FES to a stroke patient’s peroneal nerve can help improve self-confidence in balancing significantly [39]. In this study, the real-time feedback and EMG-triggered FES ankle joint training improved dynamic balance. This is because the tibialis anterior and soleus muscles essential to dynamic balance were stimulated [40]. The training included motion observations through visual feedbacks, correction, and supplementation of the stroke patient as to weight shift and symmetry [41]. These promoted the activation of the subject’s motor area of the brain and facilitated cortical reconstitution of the primary motor area [42]. The video focusing on the ankle joint motion positively influenced kinesthetic memory. The increase in the ankle joint ROM and the reduction in muscle tension led to the improvement of dynamic balance.

Foot drop as secondary damage after a stroke is attributable to insufficient or weak autonomous control of ankle and toe dorsiflexors, causing inefficient ankle dorsiflexion in the swing phase of gait and limited heel contact in the early contact phase [2]. This disturbance of normal gait patterns leads to speed reduction, confusion of weight acceptance, shifting, and unstable gait [3].

With the use of GAITRite™, this study analyzed gait changes. The RFEF group had significantly improved gait speed and cadence as temporal gait abilities. For spatial gait abilities, the RFEF group also had significantly improved step and stride lengths. Stance time and swing phase factors in the affected side of the lower limb also improved (*p* < 0.05). Except for cadence, gait variables were significantly different between groups (*p* < 0.05). In the research performed by Yom et al. (2015) [32] where they tested 20 stroke patients, the experimental group had virtual reality-based ankle motion and general physical therapy 5 times a week, for 6 weeks, whereas the control group watched a video and had general physical therapy during the same period. As a result, the experimental group significantly increased the stride length of the affected side from 68.95 to 78.27 cm (*p* < 0.05). A significant difference was noted between groups (*p* < 0.05). The experimental group significantly increased the swing phase factor of the affected side from 29.60% to 36.38%, whereas the control group significantly increased from 27.51% to 28.17% (*p* < 0.05).

In this study, the real-time feedback and EMG-triggered FES ankle joint training improved the joint ROM and reduced muscle tension, thereby improving balance. With this improvement, the gait speed, cadence, step and stride lengths of the affected side, stance time factor of the affected side, and swing phase factor of the affected side all increased. In this study, an observation video of the study subject’s motion was provided to trigger interest. The subject’s lower limb was focused during gait. Thus, even if the subject’s concentration and memory deteriorated, their concentration seemed to increase. Real-time visual feedback helped the study subject identify and correct an error of motion; this was then translated into the gait [43]. The normal adult’s ankle dorsiflexion motion in the video helped stroke patients observe motions. The real-time feedback helped them identify and correct ankle motion problems based on the visual information.

Our study had some limitation. The location of the brain lesion was identified and recorded using functional magnetic reaction imaging, but the lesion volume was not recorded.

## 5. Conclusions

Real-time feedback and EMG ankle joint training triggered a positive FES and improved ankle joint ROM, muscle tension, balance, and gait. Therefore, it is considered that this method of ankle joint training may be effective in improving the ankle joint ROM, muscle tension, balance, and gait in stroke patients.

## Figures and Tables

**Table 1:** General characteristics of the subjects (*N* = 26).

Categories	RFEF Group (*n* = 13)	EF Group (*n* = 13)	x^2^/t (*p*)
Gender (Male/Female)	13 (5/8)	13 (5/8)	0.000 (1.000)
Age (year)	57.69 ± 9.49	49.15 ± 12.80	1.931 (0.066)
Height (cm)	163.69 ± 10.26	162.46 ± 11.98	0.281 (0.781)
Weight (kg)	60.23 ± 11.27	60.85 ± 9.88	−0.148 (0.884)
BMI (kg/m^2^)	22.41 ± 3.28	23.03 ± 2.44	−0.541 (0.594)
Diagnosis			
Cerebral Hemorrhage	9 (69.2%)	8 (61.5%)	0.170 (0.680)
ICH/SAH	9/0	6/2	
Cerebral Infarction	4 (30.8%)	5 (38.5%)	
MCA/SC/CR	3/1/0	3/0/2	
Affected Side			
Right	5 (38.5%)	7 (53.8%)	0.619 (0.431)
Left	8 (61.5%)	6 (46.2%)	
Onset Time (day)	272.31 ± 90.29	274.92 ± 105.09	−0.068 (0.946)
MMSE-K (score)	27.54 ± 2.47	27.77 ± 2.58	−0.233 (0.818)

RFEF group: EMG–FES interface on ankle training combined with real-time feedback group; EF group: EMG–FES interface on ankle training group; BMI: body mass index; ICH: intracerebral hemorrhage; SAH: subarachnoid hemorrhage; MCA: middle cerebral artery; SC: striatocapsular; CR: corona radiata; MMSE-K: Mini-Mental State Examination-Korean.

**Table 2:** Differences in lower-limb function (*N* = 26).

Parameters	Trials	RFEF Group (*n* = 13)	EF Group (*n* = 13)	t (*p*)
WBLT (cm)	pretest	3.30 ± 1.70 ^a^	3.65 ± 1.67	1.896 (0.070)
	posttest	5.23 ± 2.35	4.38 ± 1.98	
	pre–post	1.92 ± 1.44	0.73 ± 0.78	−2.623 (0.015)
	t (*p*)	−4.811 (0.000)	−3.376 (0.000)	
Tardieu Scale (score)	pretest	1.35 ± 0.51	1.17 ± 0.58	0.695 (0.493)
	posttest	0.79 ± 0.63	1.06 ± 0.69	
	pre–post	−0.55 ± 0.51	−0.11 ± 0.60	59.500 (0.025)
	t (*p*)	−2.940 (0.003)	−0.357 (0.721)	

^a^ Mean ± standard deviation. RFEF group: EMG–FES interface on ankle training combined with real-time feedback group; EF group: EMG–FES interface on ankle training group; WBLT: weight-bearing lunge test.

**Table 3:** Difference on the balance (*N* = 26).

Parameters	Trials	RFEF Group (*n* = 13)	EF Group (*n* = 13)	t (*p*)
TUG (s)	pretest	36.54 ± 25.25 ^a^	20.84 ± 15.93	1.896 (0.070)
	posttest	28.73 ± 19.13	18.49 ± 13.51	
	pre–post	−7.81 ± 7.84	−2.35 ± 3.51	−2.290 (0.035)
	t (*p*)	3.593 (0.004)	2.417(0.033)	
BBS (score)	pretest	37.38 ± 9.70	40.69 ± 8.01	−0.947 (0.353)
	posttest	44.53 ± 8.51	44.69 ± 8.04	
	pre–post	7.15 ± 3.93	4.00 ± 3.08	2.275 (0.032)
	t (*p*)	−6.557 (0.000)	−4.679 (0.001)	

^a^ Mean ± standard deviation. RFEF group: EMG–FES interface on ankle training combined with real-time feedback group; EF group: EMG–FES interface on ankle training group; TUG: Timed Up and Go Test; BBS: Berg Balance Scale.

**Table 4:** Difference in gait ability (*N* = 26).

Parameters	Trials	RFEF Group (*n* = 13)	EF Group (*n* = 13)	t (*p*)
Gait velocity (cm/s)	pretest	38.85 ± 32.38 ^a^	52.47 ± 25.55	−1.190 (0.246)
	posttest	49.73 ± 37.91	56.81 ± 26.49	
	pre–post	10.88 ± 9.39	4.34 ± 5.90	2.126 (0.044)
	t (*p*)	−4.176 (0.001)	−2.651 (0.021)	
Cadence (steps/min)	pretest	69.63 ± 25.99	75.62 ± 22.74	−0.626 (0.537)
	posttest	76.60 ± 27.16	78.68 ± 23.20	
	pre–post	6.97 ± 10.26	3.05 ± 4.71	1.249 (0.229)
	t (*p*)	−2.450 (0.031)	−2.337 (0.038)	
Step length (affected) (cm)	pretest	31.83 ± 15.21	38.81 ± 10.47	−1.363 (0.185)
	post test	37.69± 17.02	40.49 ± 11.33	
	pre–post	5.86 ± 4.11	1.68 ± 4.09	2.595 (0.016)
	t (*p*)	−5.135 (0.000)	−1.479 (0.165)	
Stride length (affected) (cm)	pretest	60.01 ± 29.25	78.67 ± 21.51	−1.853 (0.076)
	posttest	70.35 ± 33.35	82.17 ± 22.31	
	pre–post	10.34 ± 6.69	3.50 ± 7.30	2.487 (0.020)
	t (*p*)	−5.566 (0.000)	−1.729 (0.109)	
Stance phase (affected) (%)	pretest	74.70 ± 7.83	72.12 ± 5.03	0.999 (0.328)
	posttest	70.30 ± 9.50	71.06 ± 4.69	
	pre–post	−4.40 ± 5.20	−1.05 ± 2.29	−2.120 (0.050)
	t (*p*)	3.050 (0.010)	1.659 (0.123)	
Swing phase (affected) (%)	pretest	25.28 ± 7.83	27.88 ± 5.03	−1.005 (0.325)
	post test	29.70 ± 9.50	28.94 ± 4.70	
	pre–post	4.41 ± 5.20	1.05 ± 2.28	2.127 (0.049)
	t (*p*)	−3.056 (0.010)	−1.669 (0.121)	

^a^ Mean ± standard deviation. RFEF group: EMG–FES interface on ankle training combined with real-time feedback group; EF group: EMG–FES interface on ankle training group.
